# Therapeutic effects of visual standard channel combined with F4.8 visual puncture super-mini percutaneous nephrolithotomy on multiple renal calculi

**DOI:** 10.12669/pjms.341.14236

**Published:** 2018

**Authors:** Zhenyu Cui, Yanjun Gao, Wenzeng Yang, Chunli Zhao, Tao Ma, Xiaoqiang Shi

**Affiliations:** 1 Zhenyu Cui, Department of Urinary Surgery, Affiliated Hospital of Hebei University, Baoding 071000, P. R. China; 2 Yanjun Gao, Department of Urinary Surgery, Affiliated Hospital of Hebei University, Baoding 071000, P. R. China; 3 Wenzeng Yang, Department of Urinary Surgery, Affiliated Hospital of Hebei University, Baoding 071000, P. R. China; 4 Chunli Zhao, Department of Urinary Surgery, Affiliated Hospital of Hebei University, Baoding 071000, P. R. China; 5 Tao Ma, Department of Urinary Surgery, Affiliated Hospital of Hebei University, Baoding 071000, P. R. China; 6 Xiaoqiang Shi, Department of Urinary Surgery, Affiliated Hospital of Hebei University, Baoding 071000, P. R. China

**Keywords:** Standard channel, Percutaneous nephrolithotomy, Multiple renal calculi

## Abstract

**Objective:**

To evaluate the therapeutic effects of visual standard channel combined with F4.8 visual puncture super-mini percutaneous nephrolithotomy (SMP) on multiple renal calculi.

**Methods:**

The clinical data of 46 patients with multiple renal calculi treated in Affiliated Hospital of Hebei University from October 2015 to September 2016 were retrospectively analyzed. There were 28 males and 18 females aged from 25 to 65 years old, with an average of 42.6. The stone diameters were 3.0-5.2 cm, (4.3 ± 0.8) cm on average. F4.8 visual puncture-assisted balloon expansion was used to establish a standard channel. After visible stones were removed through nephroscopy combined with ultrasound lithotripsy, the stones of other parts were treated through F4.8 visual puncture SMP with holmium laser. Indices such as the total time of channel establishment, surgical time, decreased value of hemoglobin, phase-I stone clearance rate and surgical complications were summarized.

**Results:**

Single standard channel was successfully established in all cases with the assistance of F4.8 visual puncture, of whom 24 were combined with a single microchannel, 16 were combined with double microchannels, and six were combined with three microchannels. All patients were placed with nephrostomy tube which was not placed in the microchannels. Both F5 double J tubes were placed after surgery. The time for establishing a standard channel through F4.8 visual puncture was (6.8 ± 1.8) min, and that for establishing a single F4.8 visual puncture microchannel was (4.5 ± 0.9) min. The surgical time was (92 ± 15) min. The phase-I stone clearance rate was 91.3% (42/46), and the decreased value of hemoglobin was (12.21 ± 2.5) g/L. There were 8 cases of postoperative fever which was relieved after anti-inflammatory treatment. Four cases had 0.5-0.8 cm of stone residue in the lower calyx, and all stones were discharged one month after surgery by *in vitro* shock wave lithotripsy combined with position nephrolithotomy, without stone streets, delayed bleeding, peripheral organ damage or urethral injury.

**Conclusion:**

Combining visual standard channel with F4.8 visual puncture SMP for the treatment of multiple renal calculi had the advantages of reducing the number of large channels, high rate of stone clearance, safety and reliability and mild complications. The established F4.8 visual puncture channel was safer and more accurate.

## INTRODUCTION

Percutaneous nephrolithotomy (PCNL) has been widely applied in the first-line treatment of complex renal calculi.^1^ With rapid development of minimally invasive technology, complex renal calculi have been gradually treated by multi-channel methods. Currently, the success rates of multi-channel PCNL, minimally invasive PCNL and super-mini PCNL have reached 64.3%~89.0%. Besides, the entire world has witnessed satisfactory clinical outcomes in increase of phase-I stone clearance rate, shortening of surgical time and decrease of number of surgeries.

Since its initial report in 2011, microperc has been increasingly used for the treatment of moderate-sized renal calculi.^2^-^6^ Complete removal of renal calculi is of great importance to the prevention of their recurrence. However, the complete recovery of complex nephrolithiasis often requires the establishment of multiple percutaneous renal channels, which may increase the risks of renal bleeding and complications. Therefore, most studies now focus on the elevation of surgical success rate and stone clearance rate by using various methods, without increasing complications.^7^ Herein, we combined standard channels of visible puncture with F4.8 visible puncture super-mini PCNL (SMP) for 46 patients with multiple renal calculi treated in our hospital between October 2015 and September 2016, with satisfactory outcomes.

## METHODS

### Baseline clinical data

This study has been approved by the ethic committee of our hospital, and written consent has been obtained from all patients. There were 46 patients, including 28 males and 18 females aged between 25 and 65 years old, 42.6 on average. In terms of clinical manifestations, there were ipsilateral low back pain in 34 cases, observable hematuria in five cases and physical examination in seven cases, with the course of disease ranging from five days to four years. The stone diameters were 3.0-5.2 cm, (4.3 ± 0.8) cm on average, including six cases of antler-like renal calculi. Besides, 29 cases had stones located on the left side and 17 cases on the right side. There were 11 cases without hydronephrosis, 27 cases with mild hydronephrosis and 8 cases with moderate hydronephrosis. The previous ESWL treatment was ineffective in 11 cases. All patients underwent preoperative blood biochemical examination, routine urine test and urine culture. Imaging examinations included kidney ureter bladder (KUB), ultrasound of urinary system (USG), and computed tomography (CT). Twenty-four patients were complicated with urinary tract infection, 11 had leukocytes of urine routines (+), 10 (++) and 3 (+++), who were preoperatively treated with sensitive antibiotics according to urinary culture until the results were negative.

### Surgical method

Epidural anesthesia was used The lithotomy position was chosen first. Zebra guide wire was placed towards the ureter on the surgical side through ureteroscopy, F5 tiger-tail urethral catheter was inserted into the ureter retrograding along the guide wire, and then the wire was pulled out. F16 Foley catheter was placed, and the two external parts were fixed to prevent prolapse. The distal end of the tiger-tail ureter catheter was connected to pressurized 0.9% saline for establishing artificial hydronephrosis. The patient was shifted to a prone position, with the renal region padded and fixed. The 11th rib tip, the 12th intercostal part, and the part between axillary line and linea scapularis were selected as the puncture site closest to the target renal calyces. A standard channel was established through visual puncture-assisted balloon dilatation. Under the guidance of ultrasound, an F4.8 needle was used to puncture the target calyx, and the path was observed through a monitor while inserting the needle. The needle core was withdrawn until the renal calculus or collection system mucosa was found. The zebra guide wire was inserted, and the skin was cut by about 1.5 cm. Then the puncture needle was withdrawn. Afterwards, the F8 fascia dilator was placed along the guide wire for pre-dilatation, and the N30 balloon dilatation catheter (BARD X-FORCE, USA) was placed through ultrasound along the guide wire to the collection system, and then connected with pressure pump, through which sterile normal saline was injected until the pressure reached 25 atm. The balloon was fully expanded for three minutes, the F24 sheath was inserted, and then the balloon dilatation catheter was removed by decompression. The nephroscope was placed to perform lithotripsy and to remove stones within the visual range using the EMS ultrasonic crush-stone system.

**Fig. 1:**
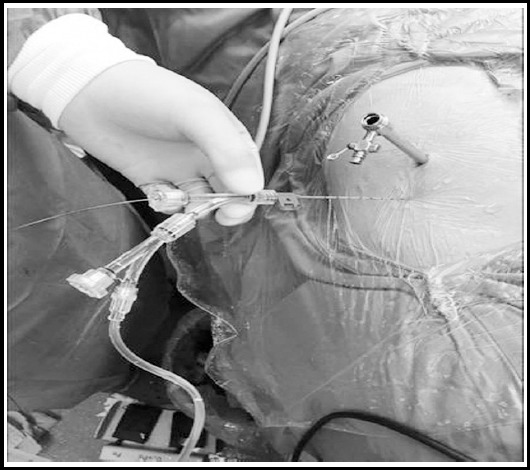
Visible standard channel combined with F4.8 visible puncture SMP.

**Fig. 2:**
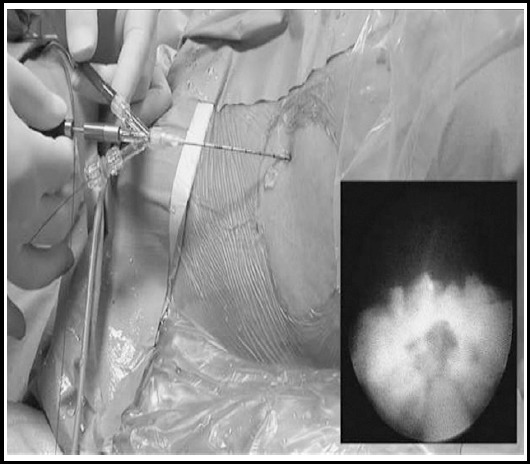
F4.8 visible puncture SMP.

**Fig. 3:**
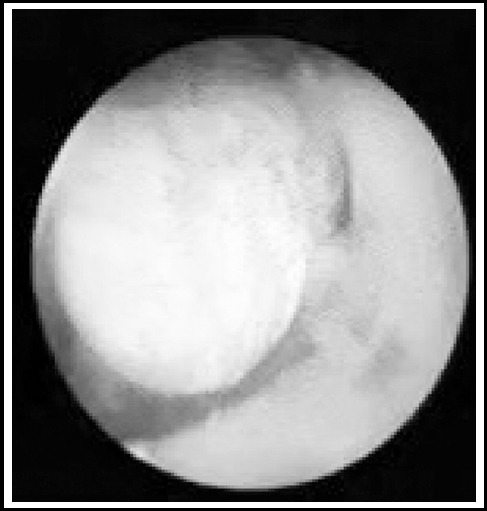
Stones were found by F4.8 visible puncture SMP.

Through intraoperative ultrasound examination, the F4.8 “visual puncture needle” was used to puncture the target calyx for the remaining stones, and the path was observed through the monitor while inserting the needle. The needle core was withdrawn until the renal calculus or collection system mucosa was found. Then the visible nephroscope system was connected, and 200 μm holmium laser fiber (frequency: 25 Hz, energy: 0.8 J) was placed to crush the stones to the size of 1-2 mm (Fig.1-3). The remaining renal calyce calculi were treated using the same method, and finally stone fragments were simultaneously sucked out via the original standard channel through nephroscope. F5 double J tubes and nephrostomy tube were retained after surgery. The tube was not retained in the microchannel. KUB or CT was rechecked one to two days after surgery to learn about stone crushing, discharge and DJ tube position. The nephrostomy tube was pulled out two to three days after surgery, and double J tubes were removed 4 to 6 weeks after surgery. If the stone fragment was larger than 4 mm, lithotripsy was performed through visual microchannel puncture combined with holmium laser at phase II one week later.

### Observation indices

We recorded indices such as the establishment time of standard channel, the establishment time of single F4.8 visible puncture microchannel, surgical time, decreased value of hemoglobin, clearance rate of phase-I stone and surgical complications.

## RESULTS

Single standard channel was successfully established in all cases with the assistance of F4.8 visual puncture, of whom 24 were combined with a single microchannel, 16 were combined with double microchannels, and six were combined with three microchannels. All patients were placed with nephrostomy tube that was not placed in the microchannels. Both F5 double J tubes were placed after surgery. The time for establishing a standard channel through F4.8 visual puncture was (6.8 ± 1.8) minutes, and that for establishing a single F4.8 visual puncture microchannel was (4.5 ± 0.9) min. The surgical time was (92 ± 15) minutes. The phase-I stone clearance rate was 91.3% (42/46), and the decreased value of hemoglobin (12.21 ± 2.5) g/L. There were 8 cases of postoperative fever which was alleviated after anti-inflammatory treatment. Four cases had 0.5-0.8 cm of stone residue in the lower calyx, in which all stones were discharged one month after surgery by *in vitro* shock wave lithotripsy combined with position nephrolithotomy, without stone streets, delayed bleeding, peripheral organ damage or urethral injury.

## DISCUSSION

Multiple renal calculi are common in clinical practice, with a low clearance rate when single-channel lithotripsy is used. It is often required to establish multi-channels or leverage lens for the treatment of antler-like renal calculi through percutaneous nephroscopy, increasing the degree of renal damage as well as the risks of serious intraoperative and postoperative complications.^8^ In recent years, with the development of minimally invasive techniques, standard-channel PCNL, minimally invasive PCNL, SMP or their combinations are often employed to treat multiple renal calculi, with the success rates of 64.3-89%.^9^-^13^

During surgery, the establishment of puncture channel is most important. When multi-channel lithotripsy is used, the angle, depth and position of the first channel directly affect the establishment of auxiliary channels. During lithotripsy, the first channel may cause changes in the structure around the kidneys due to puncture extrusion expansion, nephroscope swing, mucosal bleeding and extravasation of flushing fluid. It is more difficult to use B-guided ultrasound alone to establish a multi-channel, also posing higher requirements for the technique and experience of operating physicians. Researchers have combined retrograde flexible ureteroscopy with phase-I visual microchannel percutaneous nephroscope for the treatment of antler-like renal calculi, which used conventional method through PCNL to treat visual field stones, and then those in the upper, lower and parallel renal calyces through retrograde flexible ureteroscopy.^14^ However, this requires special positions like inclined supine lithotomy position. Most of the researchers are accustomed to choosing prone position to establish the channel, and other positions may increase the difficulty of establishment and risks. During the establishment of percutaneous renal channel, a clear field of vision should be ensured. If the field of vision of flexible ureteroscopy is obscure, there may be dual-lens interference and need for more operators. Based on the above difficulties, we combined visual standard channel with F4.8 visual puncture SMP for the treatment of multiple renal calculi.

We applied F4.8 visual puncture nephroscope system under ultrasound guidance while establishing a standard percutaneous renal channel. In the puncture process, the needle direction and trajectory could be seen on the ultrasound display screen. By using the visual puncture system, we managed to observe fat tissue, muscle tissue, perirenal fat and kidneys through which the needle passed until stone or the mucosa of the collecting system was visible, while avoiding the blood vessels and punctures being too shallow or deep, so as to lay a foundation for balloon dilatation and channel establishment. Ultrasound lithotripsy through a standard channel nephrolithotomy can quickly remove most of stones in the field of view. Residual stones are often distributed in parallel renal calyces, so soft lenses that are planted along the percutaneous renal access may increase stone clearance rate, but this technique requires a long learning curve and has significantly longer surgical time and increased risk of soft lens damage.^15^ In this case, auxiliary channels need to be established to handle the stone fragments outside the field of view of nephroscope.^16^ Akman et al.^7^ reported that the establishment of multi-channel increased the stone clearance rate, but significantly extended the surgical time and augmented the bleeding risk. Kukreja et al.^17^ found a correlation between sheath diameter and the incidence rate of bleeding. Therefore, lithotripsy of small channels is of great significance to reducing the incidence rates of bleeding and other complications. In 2011, Desai et al.^2^ combined 4.8F channel with holmium laser for lithotripsy, and referred to it as “microperc”. Clinical trials have confirmed the efficacy and safety of microperc, especially for simple subrenal calyx calculus and middle-sized renal calculi.^3^-^6^ A standard channel was used in our study to assist one or more micropercs. In the process of united lithotripsy, the standard channel was drained smoothly. When the microchannel gravels were flushed, they could flow out directly from the standard channel, thereby effectively reducing the renal pelvis pressure and keeping the visual field clear. At the same time, negative pressure suction during ultrasonic lithotripsy could also keep a low-pressure state in the renal pelvis, which could effectively prevent bacteriuria retrograde infection, reduce postoperative fever, bacteremia and other complications. In this study, the stone clearance rate in the first phase was 91.3% (42/46). Only eight patients suffered from postoperative fever which was mitigated after anti-inflammatory treatment. There was no case of major bleeding that required transfusion or interventional embolization. According to analysis on four cases of residual lower calyx stones, the reasons mainly included a larger load of stone, longer surgical time and fluid leakage which caused residual stones to be hardly observable by ultrasound.

In summary, the visual standard channel combined with F4.8 visual puncture SMP had the advantages of reducing the number of large channels, high clearance rate, safety, reliability and mild complications in the treatment of multiple renal calculi. With the application of F4.8 visual puncture, the channel establishment was safer and more targeted. Regardless, this study still has limitations, i.e. the safety and effectiveness of this approach need to be further confirmed by prospective and randomized controlled studies with larger sample sizes.

### Authors' contributions

**ZC & WY** designed this study and prepared this manuscript.

**ZC, YG, WY, CZ, TM & XS** performed this study.

**YG, CZ, TM & XS** collected and analyzed clinical data.
